# Modification of Rule of Mixtures for Tensile Strength Estimation of Circular GFRP Rebars

**DOI:** 10.3390/polym9120682

**Published:** 2017-12-07

**Authors:** Young-Jun You, Jang-Ho Jay Kim, Ki-Tae Park, Dong-Woo Seo, Tae-Hee Lee

**Affiliations:** 1 Structural Engineering Research Institute, Korea Institute of Civil Engineering and Building Technology, 283 Goyangdae-Ro, Ilsanseo-Gu, Goyang-Si 10223, Korea; ktpark@kict.re.kr (K.-T.P.); dwseo@kict.re.kr (D.-W.S.); 2 Concrete Structural Engineering Laboratory, School of Civil and Environmental Engineering, Yonsei University, 50 Yonsei-Ro, Seodaemun-Gu, Seoul 03722, Korea; jjhkim@yonsei.ac.kr (J.-H.J.K.); saintlth@yonsei.ac.kr (T.-H.L.)

**Keywords:** FRP, rebar, rule of mixtures, tensile strength, hollow section

## Abstract

The rule of mixtures (ROM) method is often used to estimate the tensile strength of fiber reinforced polymers (FRPs) reinforcing bars (rebars). Generally, the ROM method predicts the FRP rebars’ modulus of elasticity adequately but overestimates their tensile strength. This may result from defects occurred during manufacture that prevent the used materials from exhibiting a sound performance and the shear-lag phenomenon by transmission of external forces through the surface of the rebar having a circular cross section. Due to the latter, there is a difference in fiber breaking points regarding the fibers located on the surface and fibers located at the center, and thus results in differences between the values calculated from the conventional ROM and the experimental result. In this study, for the purpose of resolving the problem, glass FRP (GFRP) rebars were shaped to have a hollow section at the center of their cross sections and were further subject to tensile strength tests. The test results were further placed under regression analysis and a modified ROM within ±5% accuracy compared to the experimental value was proposed for GFRP rebars with 13, 16, and 19 mm diameters.

## 1. Introduction

Fiber reinforced polymers (FRPs) have been mainly utilized in the aeronautical, aerospace, and automotive fields owing to their relatively high strength, lightweight, and non-corroding qualities. The construction sector began paying attention to FRPs in the 1950s as they were recognized as a replacing material capable of solving the problem of degradation of structural performance caused by the corrosion of steel reinforcement in concrete structures. Accordingly, efforts were made to develop FRP reinforcing bars (rebars) in the 1960s and these efforts resulted in the major utilization of FRPs in structures in the 1970s [1]. Internationally, active research on FRP rebars in the U.S., Canada, and Europe led to the commercialization and application of several products in structure construction.

For economic reasons, glass fiber is often used rather than carbon or aramid fiber in the production of FRP rebars [2]. Even if the tensile strength of bars made with glass fiber reinforced polymers (GFRPs) attains strengths of typically around 700 MPa (ISIS 2007), recent achievements have succeeded in developing GFRP rebars with strengths higher than 1000 MPa [3,4].

If the FRP rebars were manufactured perfectly without defects and uniform load applies within the cross section, the materials used to manufacture the FRP rebars are expected to exhibit a sound performance, in which the tested tensile strength values are the same as the value calculated from previous ROM. However, due to production defects that occur as a result of the tangling of fibers or appearance of voids, the used materials may not exhibit a sufficiently sound performance [5]. In addition, in general, GFRP rebars intended to replace steel rebars have circular cross-sections with deformed or sand-coated surfaces to properly achieve the transfer of forces to the rebar in concrete. This causes the rebar to experience shear-lag where the fibers located in the periphery of the rebar cross-section receive higher stress than those located at the center. Therefore, unlike the steel rebar, the tensile strength of the FRP rebar is a function of the rebar’s diameter [6].

For this reason, the rule of mixtures (ROM) method, which evaluates the tensile strength of FRPs using linear elastic material qualities, tends to overestimate the tensile strength of FRP rebars. It is thus necessary to modify the conventional ROM method to estimate properly the tensile strength of FRP rebars.

Accordingly, this study intends to derive a ROM equation for GFRP rebars assuming that the shear-lag effect causes the stress across the rebar cross-section to be distributed along a quadratic curve when the GFRP rebar is tensioned. To that end, adjustment coefficients within ±5% accuracy compared to the experimental value are proposed based upon the tensile test results of GFRP rebars with hollow sections and diameters of 13, 16, and 19 mm.

The tensile strength performance of GFRP rebars may differ according to the type of material used (for example, E (electrical)-, S (strength)-, and AR (alkali resistant)-glass) and the method by which they were manufactured. Today, several different types of GFRP rebars have been developed and there exist structural design guidelines regarding the application of some products. However, the development of GFRP rebars is currently a work in progress. In light of this, despite the results of this study not being appropriate for application to all GFRP rebars, the GFRP rebars considered in this research were used to propose a modified ROM that considers only one variable, which was focused on the sound performance of the fiber used in the GFRP rebar (relatively higher tensile strength/fiber content than previously researched GFRP rebars). Due to the linear material feature, this model may help other researchers as an index in the design and evaluation of GRFP rebar through any method like linear interpolation.

## 2. Background

The rule of mixtures (ROM) is a weighted mean used to predict the properties of composite materials such as FRPs including the tensile performance based upon the following assumptions [7]:

(1) One ply is microscopically homogenous, linear elastic, and orthotropic. In addition, it is initially in a stress-free state.
(2) The fiber is homogenous, linear elastic, and well-arranged regularly in space.
(3) The matrix is also homogenous, linear elastic, and isotropic.
(4) There are no voids, and the fiber and the matrix are completely coupled.

Based on these assumptions, the tensile performance of FRP composed of fiber and polymer matrix can be obtained by combining linearly the volume fraction and the tensile properties of the fiber and the matrix as follows [8]:

(1) $$\sigma_{FRP}=\sigma_{f}V_{f}+\sigma_{m}V_{m}$$

(2) $$E_{FRP}=E_{f}V_{f}+E_{m}V_{m}$$

where $\sigma_{FRP}$, $\sigma_{f}$, and $\sigma_{m}$, and $E_{FRP}$, $E_{f}$, and $E_{m}$ indicate the tensile strength (MPa) and the elastic modulus (MPa) of FRP, reinforcing fiber, and matrix, respectively. $V_{m}$ and $V_{f}$ are the volume fractions (%) of the reinforcing fiber and matrix, respectively.

ROM is the simplest and easiest method to predict the tensile properties of FRP with a given fiber volume fraction and using the characteristics of its components. However, although its prediction of the tensile elastic modulus in the axial direction is effective or accurate, ROM fails to predict accurately the tensile strength [9]. In addition, ROM assumes that the fiber is unidirectionally aligned and stress is uniformly distributed. In reality, the spread of the fiber can be non-homogenous and the fiber orientation can be misaligned, resulting in reduced tensile strength of the unidirectional fiber composite [10].

**Figure 1:**
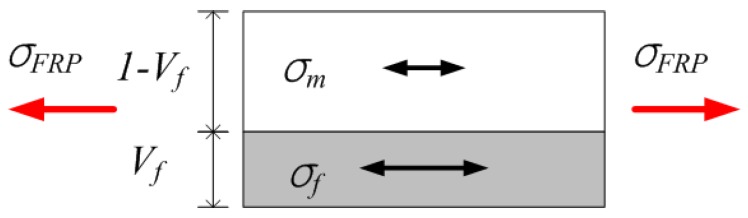
Schematic illustration of deformation of FRP under stress [9].

Moreover, ROM also considers that the fiber and the matrix experience identical deformation, as shown in Figure 1. However, since both materials exhibit different tensile behaviors, shear-lag occurs and causes rupture to occur at different points of time.

**Figure 2:**
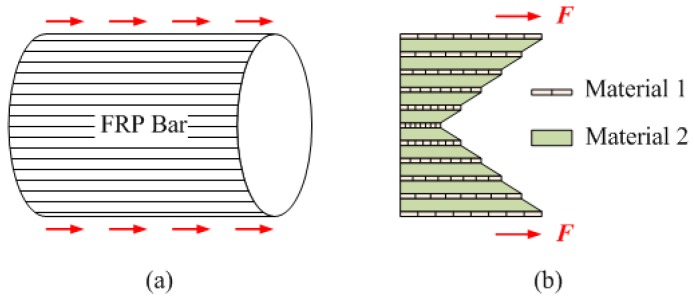
Shear-lag phenomenon: (**a**) overview; and (**b**) longitudinal section.

When a rebar is tensioned by grips, shear-lag will not occur when the bar is made of a highly stiff material such as steel. However, as shown in Figure 2, this is not the case for FRP which combines materials with relatively high stiffness (fiber) and relatively low stiffness (resin). This explains why ROM predicts the elastic modulus of FRP rebars with relatively good accuracy but overestimates the tensile strength.

Accordingly, Lee and Hwang [10] proposed the modified ROM expressed in Equations (3) and (4), in which an effective fiber volume fraction is applied by means of a degradation parameter. The equations are based on the assumption that the ROM-originated strength variation is affected by factors such as the non-homogenous fiber spread in the case of a small fiber volume fraction and the lack of a matrix between some adjacent fibers.

(3) $$\sigma_{FRP}=\sigma_{f}V_{fe}+\sigma_{m}(1-V_{f})$$

(4) $$V_{fe}=V_{f}(1-P)$$

where $V_{fe}$ is the effective volume fraction of reinforcing fiber (%) and P is a degradation parameter. Other symbols are same as those in Equations (1) and (2).

**Figure 3:**
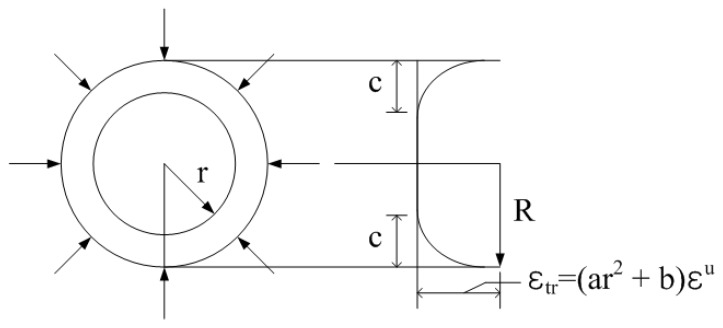
Strain distribution across cross-section of FRP rebar in tension [11].

Faza and Gangarao [11] developed an analytical model to estimate the tensile strength of FRP rebars based on the mechanics of materials. This model considered the shear-lag effect by assuming that the strain of the bar resulting from the tension introduced by the gripping system is parabolic and axisymmetric in the circular cross-section as shown in Figure 3. The parameter c denoting the thickness of the boundary layer was introduced to relate the curing rates associated with the rebar’s size. To establish their model expressed in Equation (5), the authors assumed the strain distribution shown in Figure 3. In this distribution, the strain diminishes parabolically from the outermost part of the rebar to the height of D/2−c, where D is the diameter of the FRP rebar, and shows even distribution afterward to the center with the fiber’s extreme strain decreased by λ.

(5) $$\sigma_{FRP}=0.5\sigma_{f}[1+2\lambda -{\left(1-2c/D\right)}^{2}]$$

Equation (5) predicts the actual tensile strength of the FRP rebars quite well when adequate values are used for the parameters. However, the determination of the two parameters c and λ depends on the curing rate of the resin and the size of the specimens, respectively.

Despite it being practical to consider performance losses due to incomplete bonds between fibers, voids within the composite, and the non-homogeneous spread of fibers, such elements of incompletion for cases of relatively small bar shaped diameters and long lengths may have very little effect, or an extremely large effort is needed to quantify all such elements of incompletion.

## 3. Model for Tensile Strength Estimation

The tensile strength of the FRP rebars is sensitive not only to the amount and material properties of the fibers but also to various other conditions such as the manufacturing process and fiber arrangement [5]. Moreover, since reinforced fibers are highly vulnerable to lateral loading, the tensile strength of the FRP rebars may differ even according to the grip system used to apply tension as well as to the bond performance between the grip and the FRP rebar surface [12,13].

Considering the difficulty of taking into account all such manufacturing conditions, the following preconditions were assumed macroscopically in this study to develop an equation predicting the tensile strength of FRP rebars.

(1) FRP rebars are made only of a single type of reinforced fiber (especially glass fiber) and resin.
(2) FRP rebars are fabricated through an adequate process, and the so-produced rebars exhibit small variation regarding the tensile strength.
(3) Because the values of material properties of resin are far smaller than those of reinforcing fiber, it is assumed that, for the sake of model simplification, the tensile strength of the FRP rebars is controlled macroscopically only by the quantity of the reinforcing fiber.
(4) There is no transverse shrinkage perpendicular to the longitudinal axis of the rebar.
(5) The grip used to evaluate the tensile strength of the FRP rebars supports the rebars sufficiently up to their breaking load or displays the same performance.
(6) The conventional ROM predicts accurately enough the elastic modulus of FRP rebars.

The equation predicting the tensile strength of the FRP rebars proposed in this research resembles that suggested by Faza and Gangarao [11]. However, considering that the diameter and length of the rebars are sufficiently comparable, the equation is formulated by ignoring the section where the strain is distributed evenly and by assuming that the distribution of stress occurs inside the cross-section as the quadratic curve in Equation (6).

**Figure 4:**
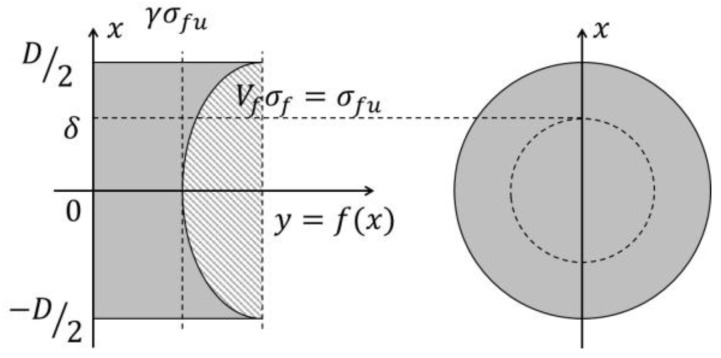
Assumed stress distribution on transversally projected section.

If the distribution of the stress developed in the FRP rebars under tension and close to rupture is axisymmetric and takes the form of a quadratic curve, the stress distribution across the rebar cross-section can be expressed as shown in Figure 4.

(6) $$f(x)=Ax^{2}+Bx+C$$

In Figure 4, $\sigma_{fu}$ is the ultimate tensile strength of FRP depending on the fiber volume fraction ($V_{f}$); $\sigma_{f}$ and D are the tensile strength and the diameter of a rebar, respectively; δ is a radius of any point from the center (which means the radius of hollow section in this paper); and γ is a reduction factor of the tensile strength (0 < γ < 1).

**Figure 5:**
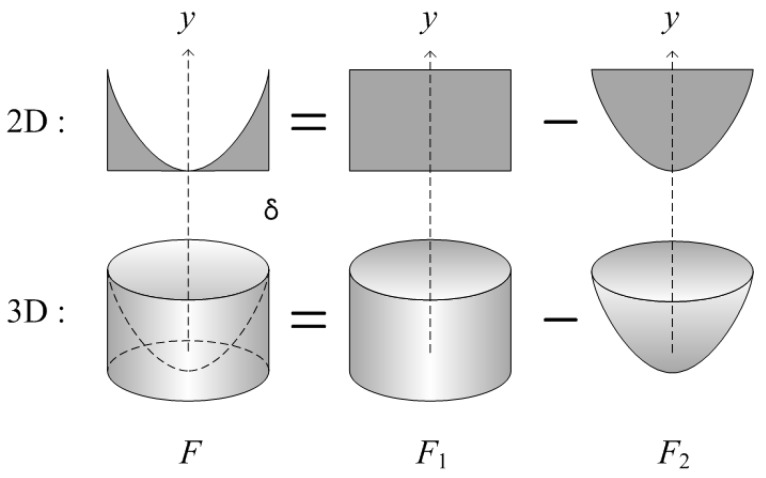
Calculation of peak tensile force.

The size of the distributed stress can be obtained by subtracting the volume of the paraboloid bowl from the circular cylinder in Figure 5. The volume is computed as the height of the stress multiplied by the area and means thus the force.

In this stress distribution, the ultimate tensile strength ($\sigma_{fu}$) occurs across the rebar’s outer surface while the ultimate tensile strength reduced by γ is at work in the center of the cross-section. Therefore, the quadratic curve projected onto the side passes through (0, $\gamma \sigma_{fu}$), (*D*/2, $\sigma_{fu}$), and (−*D*/2, $\sigma_{fu}$), leading to $C=\gamma \sigma_{fu}$, B=0 and $A=(1-\gamma )\sigma_{fu}/{\left(D/2\right)}^{2}$.

In Figure 4 and Figure 5, the volume $F_{1}$ of the cylinder with radius δ and height f(δ), and the volume $F_{2}$ of the paraboloid bowl can be obtained as follows:

(7) $$F_{1}=\pi \delta^{2}f(\delta )=\pi \delta^{2}\left\{\frac{(1-\gamma )\sigma_{fu}}{{\left(D/2\right)}^{2}}\delta^{2}+\gamma \sigma_{fu}\right\}$$

(8) $$F_{2}=\pi \int_{\gamma \sigma_{fu}}^{f(\delta )}x^{2}dy=\frac{\pi }{2}\frac{(1-\gamma )\sigma_{fu}}{{\left(D/2\right)}^{2}}\delta^{4}$$

(9) $$F=F_{1}-F_{2}$$

Considering the FRP rebar with radius δ and hollow part in the cross, as shown in Figure 5, the volume $F^{Empty}$ of the removed part of the composite volume can be obtained by Equation (9), and the volume of the full cross-section $F^{Full}$ can also be obtained by Equation (9) in which δ in Equations (7) and (8) is replaced by *D*/2. That is,

(10) $$F^{Empty}=F_{1}^{Empty}-F_{2}^{Empty}=\frac{\pi }{2}\delta^{2}\sigma_{fu}\left\{\frac{(1-\gamma )\sigma_{fu}}{{\left(D/2\right)}^{2}}\delta^{2}+2\gamma \right\}$$

(11) $$F^{Full}=\pi {\left(\frac{D}{2}\right)}^{2}\sigma_{fu}\left(\frac{1+\gamma }{2}\right)=\left(\frac{1+\gamma }{2}\right)A_{FRP}V_{f}\sigma_{fu}$$

where $A_{FRP}$ is the cross-sectional area of the FRP rebar.

Consequently, Equation (11) representing the tensile breaking force of the rebar is a modified ROM because it multiplies Equation (1) by the cross-section area of the rebar and applies the tensile strength reduction rate.

Assuming that, with the exception of the reduced tensile strength, the tensile behavior of the FRP rebar with a hollow section is not different from that of the FRP rebar with a full cross section in terms of the elastic modulus, the tensile strength of the hollow section FRP rebar can be obtained as follows.

(12) $$F^{Hollow}=F^{Full}-F^{Empty}=\left(\frac{1+\gamma }{2}\right)A_{FRP}V_{f}\sigma_{fu}-\frac{\pi }{2}\delta^{2}\sigma_{fu}\left\{\frac{(1-\gamma )\sigma_{fu}}{{\left(D/2\right)}^{2}}\delta^{2}+2\gamma \right\}$$

The tensile strength of the FRP rebar can be obtained adequately, if the tensile strength change rate γ can be obtained from Equation (12) through tension test of the hollow FRP rebar with parts missing in the center of the cross-section.

Unlike the previous ROM model that assumes the external force to be equally distributed across the FRP cross-section, this model features a bar shaped FRP having a circular cross-section that considers actual situations in which external forces are not transmitted to the cross-section but from the side section surface. Due to there being only one variable, it is easier to apply than Equation (5) that uses two variables.

## 4. Tensile Test of Hollow GFRP Rebar Specimens

### 4.1. Materials and Manufacturing Process

The GFRP rebars used in this study are made of E-glass fiber (Owens Corning Korea, Seoul, Korea) and vinyl ester resin (Aekyung Chemical Co., Ltd., Seoul, Korea, Ashland Inc., Covington, KY, UAS). The properties of these materials by manufacturers are listed in Table 1.

The rebars were fabricated by the braidtrusion process of You et al. [5], which is a modified version of the one suggested by Ko et al. [14]. This modified process enhances the overall tensile performance through improved fiber arrangement and reduced voids by applying a definite tension to the fibers used to form reinforced fiber bundles and ribs.

### 4.2. Hollow GFRP Rebar Specimens

Sets of GRFP rebars with two different diameters and four different hollowness ratios were fabricated to investigate the changes in the tensile properties according to the hollowness ratio of the GFRP rebars. Plain GFRP rebars (in other words, rebars with solid cross-section) were also considered to provide reference specimens for the comparison of the tensile properties with respect to the hollowness ratio. The hollow GFRP rebars were manufactured by inserting tubes with different diameters inside the rebars. Table 2 lists the considered specimens with their designation, specifications, and quantities.

In the specimen designation of Table 2, D followed by a two-digit number denotes the outer diameter of the GFRP rebar, and HD indicates the diameter of the hollow tube inserted inside the GFRP rebar. For instance, D19HD8 designates the specimen with a 19-mm diameter for the whole GFRP rebar cross-section in which a hollow tube with an external diameter of 8 mm is inserted. The diameter of the reinforcing bars in Table 2 is measured at parts without ribs.

**Figure 6:**
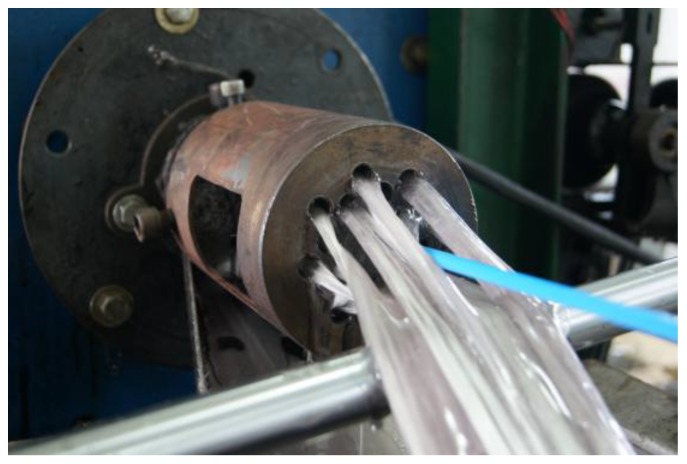
Tube insertion for hollow section.

**Figure 7:**
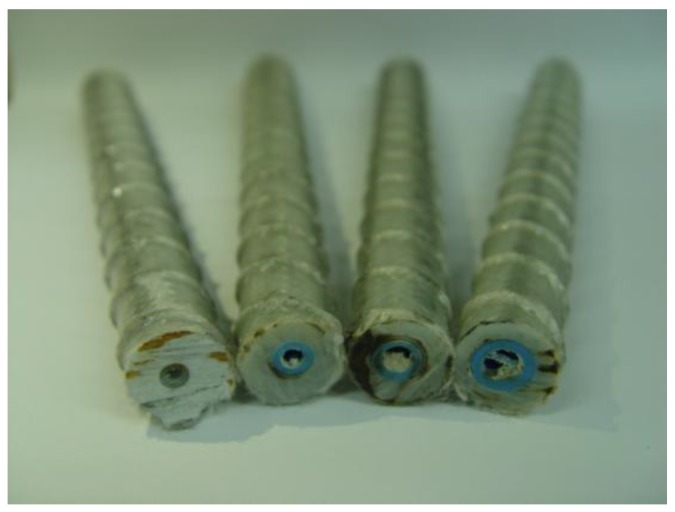
Cross-section of hollow GFRP rebars (D16).

Polyurethane tubes were used to form the hollow section within the GFRP rebar cross section. As shown in Figure 6, a fixture was used to control the centrality of the tube inside the glass fiber bundles. Figure 7 shows the cross-sections of the so-fabricated hollow GFRP rebars.

**Figure 8:**
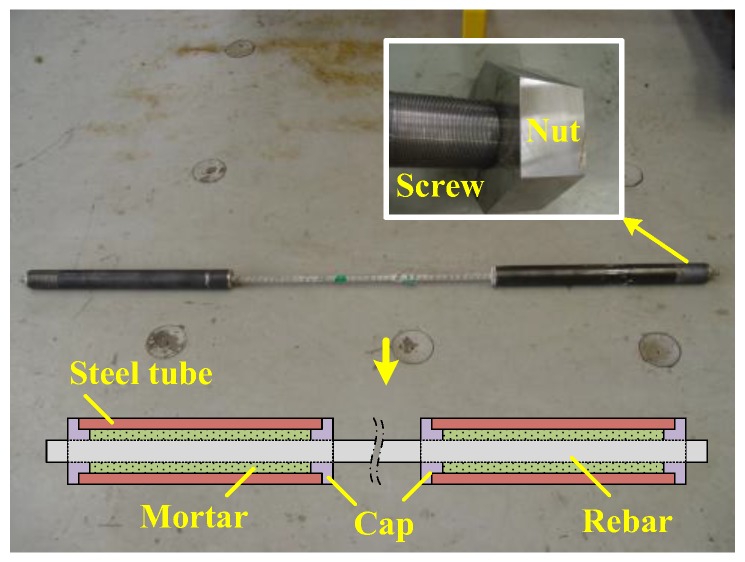
Preparation of specimens for tensile test.

The finished GFRP rebars were cut to a definite length, and cylindrical steel pipes were positioned at the ends to form the grip in compliance with CSA [15]. These steel pipes have respective thickness and length of 5.1 mm and 700 mm for the D16 rebars, and 7.1 mm and 1000 mm for the D19 rebars. The caps placed on both ends of the steel pipes are hollowed at their center to allow the rebar be inserted centrally in the steel pipes. The grips were first made on one side of the rebar and the space between the steel pipe and the rebar was filled with non-shrinkage mortar. The rebars were placed straight by means of holders and curing was conducted for seven days. Figure 8 shows a sample of GFRP rebar specimens prepared for tensile test.

### 4.3. Test Set-Up

**Figure 9:**
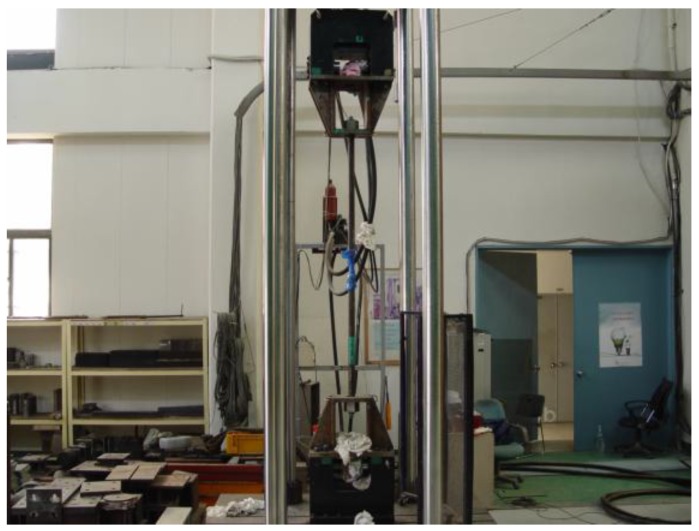
Tensile test set-up.

As shown in Figure 8 and Figure 9, one end of the cylindrical steel pipe forming the grip was threaded to fasten a nut to hold the specimen and apply tension on the two ends of the grip.

The so-prepared specimen was then installed in a universal testing machine (UTM) with capacity of 1000 kN and an electrical resistance strain gauge was attached to the middle of the rebar’s length to measure the strain during test. The place where the strain gauge was attached was cleaned softly, not grinded because the loss of cross-section would affect the tensile capacity. Tensile test was performed through displacement control and a data logger (TDS530 produced in Tokyo Sokki Kenkyujo Co., Ltd., Tokyo, Japan) was used to record the load and corresponding strain.

## 5. Results

### 5.1. Tensile Behavior of GFRP Rebar

**Figure 10:**
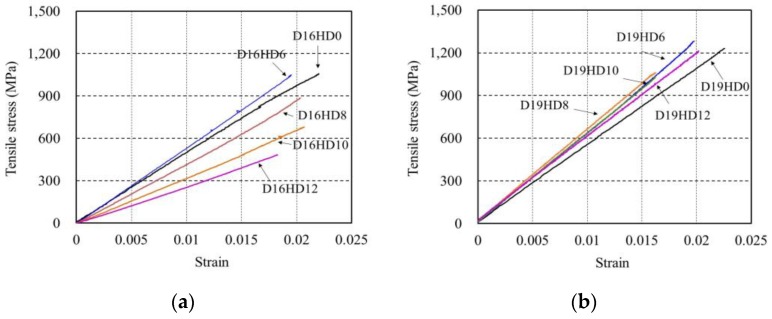
Tensile behavior of specimens: (**a**) D16; and (**b**) D19.

Figure 10 plots the tensile stress–strain curves in which the stress is the UTM’s load divided by the area of specimen and the strain is the one measured at the middle of the rebar’s length. The curves indicate a linear relationship between the tensile strength and the strain curves, and the occurrence of sudden rupture at the peak load. This is the typical material behavior of FRP where the linear increase is followed by brittle failure. The maximum tensile strength and the elastic modulus change with respect to the hollowness ratio, and tend to decrease with a larger hollowness ratio. Table 3 lists the maximum tensile strength and elastic modulus of the considered specimens. The maximum tensile strengths were calculated from dividing measured maximum loads by solid cross-section areas without considering the hollowness because the value calculated from net cross-section area with considering the hollowness means the tensile strength of a bar with smaller diameter and solid section (for example, the net area of a bar with 16 mm and 9.7 mm of outer and hollow diameters, respectively, is same with the solid area of a bar with 12.7 mm diameter). The measured average diameters of the specimens were all 16.0 mm, except for D16HD0 (15.9 mm). It appears that, apart from specimens D16HD8 and D16HD12, the coefficient of variation (C.O.V) obtained by dividing the standard deviation (S.DEV) by the mean remains below 6.4% and indicates consistency among the experimental values.

### 5.2. Regression Analysis

Regression analysis can be performed easily by the least squares method because the assumed tensile stress distribution curve expressed in Equation (6) includes one independent variable and one dependent variable. The resulting regression equation must be verified for each specimen to determine its agreement with the measured values. This can be done using the coefficient of determination $R^{2}$ of which value runs between 0 and 1, with $R^{2}$ = 1 indicating that the sample regression line fits perfectly with the data.

The reduction factor γ of the tensile strength can be obtained by regression analysis of the values in Table 3 and Equations (1) and (6)–(12). The results are γ = 0.31843 for D16 specimens and γ = 0.29549 for D19 specimens. Applying these values in Equation (11) leads to the following estimation of the tensile strength in Equation (13) for D16 specimens and Equation (14) for D19 specimens.

(13) $$F_{D16}^{Full}=0.659A_{FRP}V_{f}\sigma_{f}$$

(14) $$F_{D19}^{Full}=0.648A_{FRP}V_{f}\sigma_{f}$$

**Figure 11:**
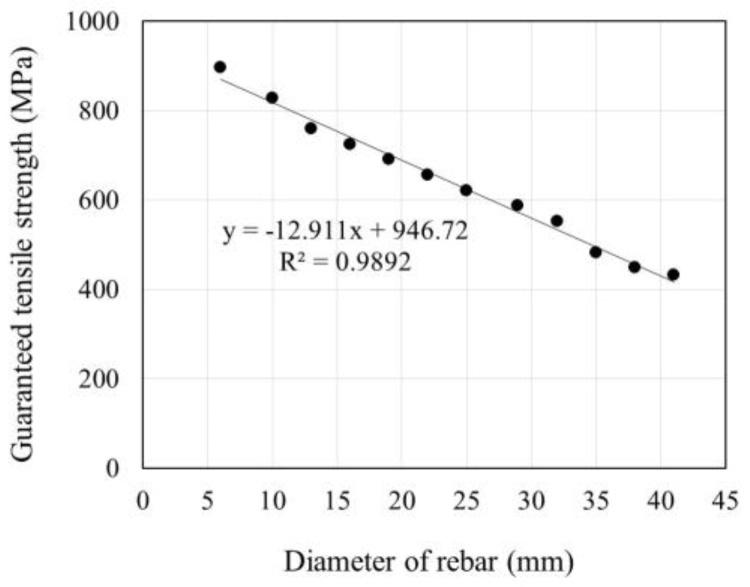
Change in tensile strength of GFRP rebar according to diameter [16].

As explained above, the tensile strength of the FRP rebars is a function of the rebar diameter and tends to decrease with a larger diameter. Figure 11 indicates the change in the tensile strength according to the diameter for the commercial product Aslan100 [16]. It appears that the tensile strength of the reinforcing bar reduces quasi-linearly with the increase of the diameter.

Accordingly, assuming a linear relationship between the tensile strength and the diameter of the GFRP reinforcing bar, the tensile strength can be estimated by Equation (15) for the 13 mm-diameter rebar through linear interpolation of Equations (13) and (14).

(15) $$F_{D13}^{Full}=0.674A_{FRP}V_{f}\sigma_{f}$$

### 5.3. Verification of Estimation Model

Although the actual fiber content is unknown because burn-out test was not performed on the specimens considered in this research, many researchers reported that the ROM provides adequate predictions of the elastic modulus of FRP materials. Thus, if the influence of the resin is ignored, as in this study, the fiber content listed in Table 4 can be estimated by Equation (2) and by ignoring the contribution of the matrix to the tensile strength.

Table 5 compares the tensile strengths predicted by the conventional and modified ROMs to validate the developed estimation model using the experimental results of this study and those of previous tensile strength tests carried out by other researchers. It appears that the modified ROM provides accuracy within ±5%, whereas the conventional ROM overestimates the tensile strength of the GFRP reinforcing bars by more than 40%.

## 6. Conclusions

Considering that the conventional ROM overestimates the tensile strength of FRP rebars with circular cross-section, this study proposed modified ROM coefficients based upon the tensile strength test results obtained for a series of GFRP rebars with various diameters and hollowness ratios. According to the type and volume of the materials used, FRP bars each present a different performance, thus it is not appropriate to extrapolate from this model and apply the results generally. However, the results of this study are expected to be applicable for use as an index for design and evaluation purposes by researchers developing GFRP rebars. The conclusions found through this research are as follows.

(1) For a specific composite that is bar shaped and has a circular cross-section, a more realistic phenomenon is realized, whereby the tensile force is transferred not through the cross-section but the side surface of the bar and this resulting uniform stress distribution is included in the proposed ROM.
(2) The proposed ROM has just one parameter to explain the shear-lag effect of the FRP bar under tension and the parameter was determined from tensile test results for GFRP rebars with a hollow section since the fiber content is the main factor for the tensile performance of a FRP outlined in the conventional ROM.
(3) The proposed ROM provides accuracy within ±5%, whereas the conventional ROM overestimates the tensile strength of the GFRP reinforcing bars with diameters of 13, 16, and 19 mm by more than 40%.

## Figures and Tables

**Table 1:** Material properties of GFRP (glass fiber reinforced polymers) rebar.

Material	Designation of Product	Tensile Strength (MPa)	Elastic Modulus (MPa)
Resin 1	HETRON 922	86	3170
Resin 2	DION-9100	79	3216
Core fiber	SE1200	2600	81,000

**Table 2:** Test specimens.

Specimen	Quantity	Diameter (mm)	Ratio of Hollow Section	Number of Roving
D16HD0	5	15.9	-	158
D16HD6	6	16.0	14.1%	129
D16HD8	6	16.0	25.0%	112
D16HD10	6	16.0	39.1%	91
D16HD12	6	16.0	56.3%	65
D19HD0	3	18.6	-	217
D19HD6	6	18.6	10.4%	196
D19HD8	6	18.7	18.3%	180
D19HD10	6	18.8	28.4%	159
D19HD12	6	19.0	39.7%	133

**Table 3:** Tensile test results of plain and hollow GFRP rebar specimens.

Specimen and Values	Peak Load (N)	Tensile Strength (MPa)	Elastic Modulus (MPa)	Specimen and Values	Peak Load (N)	Tensile Strength (MPa)	Elastic Modulus (MPa)
D16HD0	213,191	1074	49,402	D19HD0	330,210	1218	53,520
	209,767	1056	46,798		333,300	1229	55,180
	213,848	1077	50,681		306,760	1131	56,519
mean	212,269	1069	48,960	mean	323,423	1193	55,073
S.DEV.	2191	11	1979	S.DEV.	14,513	54	1502
C.O.V	1.0%	1.0%	4.0%	C.O.V	4.5%	4.5%	2.7%
D16HD6	217,120	1080	46,916	D19HD6	312,530	1147	55,623
	211,250	1051	53,008		301,480	1106	56,681
	203,650	1013	46,237		308,260	1131	57,229
	221,400	1101	51,907		280,370	1029	55,190
	207,710	1033	47,131		304,420	1117	57,505
	202,160	1005	-		277,080	1016	59,276
mean	210,548	1047	49,040	mean	297,357	1091	56,917
S.DEV.	7582	38	3161	S.DEV.	14,938	55	1464
C.O.V	3.6%	3.6%	6.4%	C.O.V	5.0%	5.0%	2.6%
D16HD8	176,830	879	42,314	D19HD8	236,600	863	52,641
	178,360	887	41,436		246,140	898	49,577
	176,520	878	40,710		248,740	908	50,267
	179,780	894	49,002		230,930	843	54,674
	181,870	905	41,146		239,260	873	50,036
	172,470	858	-		-	-	-
mean	177,638	884	42,922	mean	240,334	877	51,439
S.DEV.	3213	16	3449	S.DEV.	7209	26	2163
C.O.V	1.8%	1.8%	8.0%	C.O.V	3.0%	3.0%	4.2%
D16HD10	131,050	652	35,680	D19HD10	205,860	746	49,664
	133,670	665	33,663		199,220	722	46,453
	146,530	729	34,246		203,320	736	44,988
	137,170	682	31,801		203,690	738	45,080
	133,060	662	34,370		-	-	-
	129,790	646	37,302		-	-	-
mean	135,212	672	34,510	mean	203,023	735	46,546
S.DEV.	6094	30	1861	S.DEV.	2772	10	2184
C.O.V	4.5%	4.5%	5.4%	C.O.V	1.4%	1.4%	4.7%
D16HD12	86,330	429	28,130	D19HD12	194,500	684	38,819
	89,500	445	29,762		207,820	731	35,442
	97,260	484	26,079		182,040	640	34,792
	92,350	459	26,601		207,730	730	33,410
	83,030	413	22,041		189,390	666	37,175
	90,580	451	25,959		189,740	667	32,999
mean	89,842	447	26,429	mean	195,203	686	35,439
S.DEV.	4914	24	2593	S.DEV.	10,521	37	2233
C.O.V	5.5%	5.5%	9.8%	C.O.V	5.4%	5.4%	6.3%

S.DEV: standard deviation; C.O.V: coefficient of variation.

**Table 4:** Calculation of fiber volume fraction.

Rebar	Measured Inner Diameter (mm)	Elastic Modulus (GPa)		Calculated Volume Fraction
		Glass Fiber ^a^	Rebar ^b^	
D13 [5]	12.7	73	49	67.6%
D13 [17]	12.7	81	52	63.8%
D13 [18]	12.5	-	47	55.0%
D16	16.0	81	49	60.4%
D19 [19]	18.6	73	47	64.0%
D19	18.6	81	55	68.0%

^a^ Provided by manufacturer; ^b^ Measured experimentally.

**Table 5:** Comparison of tensile strengths predicted by conventional ROM and modified ROM using Equations (13)–(15).

Rebar	Tensile Strength (MPa)		Modification Factor	Predicted Tensile Strength (MPa)			
	Glass Fiber ^a^	Rebar ^b^		ROM *	ROM/Experiment	Modified ROM	Modified ROM/Experiment
D13 [5]	2580	1132	0.674	1745	154%	1174	104%
D13 [17]	2600	1103	0.674	1660	150%	1117	101%
D13 [18]	2600	975	0.674	1430	147%	965	99%
D16	2600	1069	0.659	1572	147%	1036	97%
D19 [19]	2580	1030	0.648	1651	160%	1069	104%
D19	2600	1193	0.648	1768	148%	1145	96%

* From Equations (1) and (2).
